# Mutations in the IGF-II pathway that confer resistance to lytic reovirus infection

**DOI:** 10.1186/1471-2121-5-32

**Published:** 2004-08-27

**Authors:** Jinsong Sheng, Edward L Organ, Chuanming Hao, K Sam Wells, H Earl Ruley, Donald H Rubin

**Affiliations:** 1 Research Medicine, Veterans Affairs Tennessee Valley Healthcare System, Nashville, TN, 37212, USA; 2 Department of Medicine, Division of Infectious Diseases, Vanderbilt University, Nashville, TN, USA; 3 Department of Microbiology and Immunology, Vanderbilt University, Nashville, TN, 37232, USA; 4 Department of Medicine, Division of Nephrology, Vanderbilt University, Nashville, TN, 37232, USA; 5 Department of Molecular Physiology and Biophysics, Vanderbilt University, Nashville, TN, 37232, USA

## Abstract

**Background:**

Viruses are obligate intracellular parasites and rely upon the host cell for different steps in their life cycles. The characterization of cellular genes required for virus infection and/or cell killing will be essential for understanding viral life cycles, and may provide cellular targets for new antiviral therapies.

**Results:**

A gene entrapment approach was used to identify candidate cellular genes that affect reovirus infection or virus induced cell lysis. Four of the 111 genes disrupted in clones selected for resistance to infection by reovirus type 1 involved the insulin growth factor-2 (IGF-II) pathway, including: the mannose-6-phosphate/IGF2 receptor (*Igf2r*), a protease associated with insulin growth factor binding protein 5 (*Prss11*), and the CTCF transcriptional regulator (*Ctcf*). The disruption of *Ctcf*, which encodes a repressor of *Igf2*, was associated with enhanced *Igf2 *gene expression. Plasmids expressing either the IGF-II pro-hormone or IGF-II without the carboxy terminal extension (E)-peptide sequence independently conferred high levels of cellular resistance to reovirus infection. Forced IGF-II expression results in a block in virus disassembly. In addition, *Ctcf *disruption and forced *Igf2 *expression both enabled cells to proliferate in soft agar, a phenotype associated with malignant growth *in vivo*.

**Conclusion:**

These results indicate that IGF-II, and by inference other components of the IGF-II signalling pathway, can confer resistance to lytic reovirus infection. This report represents the first use of gene entrapment to identify host factors affecting virus infection. Concomitant transformation observed in some virus resistant cells illustrates a potential mechanism of carcinogenesis associated with chronic virus infection.

## Background

Viruses as obligate intracellular parasites rely upon the host cell for different steps in their life cycle, including attachment, disassembly, transcription, translation, reassembly, and egress. Consequently, characterization of these cellular processes will be essential for any understanding of viral life cycles, and may provide cellular targets for new antiviral therapies.

The susceptibility to virus infection varies greatly among different cell types, and virus-resistant cells frequently emerge post-infection [1-4]. This suggests that host cell contributions to the virus life cycle, although complex, have genetic determinants. We therefore used a genetic approach to identify cellular genes required for infection by reovirus, a small cytolytic RNA virus that replicates in the cytoplasm. A gene trap retrovirus was use to create libraries of rat intestinal epithelial (RIE-1) cell clones in which each clone contained a single gene disrupted by an integrated retrovirus. The mutant libraries were then infected with reovirus, and resistant clones were selected. We hypothesized that genes mutated by gene entrapment may confer reovirus resistance as a result of either haploinsufficiency or loss of heterozygosity and could be identified by characterizing the genes disrupted by the entrapment vector. From these experiments we have isolated 152 clones and have characterized mutations in 111 different genes, providing potential candidates required for reovirus infection and/or cell killing. Many of the disrupted genes have known or imputed functions, and several are known to function in the same or related pathways. For example, four mutations affected genes in the insulin growth factor-2 (IGF-II) pathway, including genes encoding the IGF-ll/manose-6-phosphate receptor [5,6] (*Igf2r*, locus ID 25151), the IGF binding protein 5 protease [7] (*Prss11*, locus ID 65164, 2 clones), and CTCF (*Ctcf*, locus ID 83726), a transcriptional repressor of the IGF-II gene (*Igf2*) involved in paternal imprinting.

The frequency of mutations involving the IGF-II pathway led us to investigate the role of IGF-II in reovirus infection. Clone 6B72, which contains a mutation in *Ctcf *[8], was found to over express *Igf2 *transcripts, consistent with the known role of CTCF as a transcriptional repressor of the *Igf2 *gene. Moreover, forced expression of IGF-II in RIE-1 cells was sufficient to confer cellular resistance to lytic reovirus infection. Enforced IGF-II expression also transformed RIE-1 cells to anchorage independent growth, a phenotype associated with malignant change. These results represent the first use of gene entrapment to identify components of host cell metabolism required for virus infection and illustrate a potential mechanism of carcinogenesis associated with chronic virus infection.

## Results

### Disruption of the CTCF gene results in cells resistant to lytic infection by reovirus

**Figure 1:**
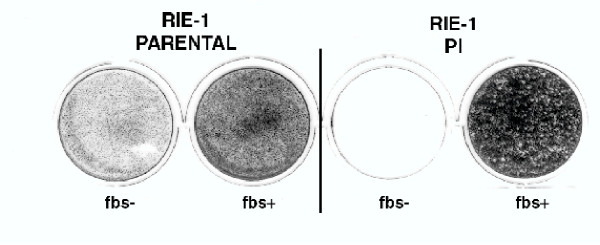
**Persistently infected RIE-1 cells fail to survive in serum-free media. **RIE-1 parental cells and cells persistently infected with reovirus type 1 were plated in complete medium (FBS^+^) or in media in which the serum was omitted (FBS^-^). Surviving cells were stained with gentian violet after 7 days. Darkly staining wells represent cell survival.

Gene entrapment strategies to identify host genes required virus replication depend on methods to select for virus resistant clones present at about one in 10^4^–10^5 ^mutagenized cells. Unfortunately, cells persistently infected with reovirus (PI) can emerge at high frequencies (one in 10^2^–10^3^) and are intrinsically resistant to the virus [9,10]. In preliminary studies, we found that hepa 1/a cells were not suitable for genetic studies due to the emergence of PI clones. However, persistently infected rat intestinal epithelial cells (RIE-1) [11,12] were found to require a serum survival factor and die when placed in serum free media (Figure 1). *In vitro *infection of RIE-1 cells with reovirus also appears to mirror virus replication in the rodent host [13-15]. Consequently, RIE-1 cells were used in the present study, and reovirus-resistant clones were selected in serum-free medium to remove PI survivors. RIE-1 cells were mutagenized by using the U3NeoSV1 gene trap shuttle vector [16] (see Methods) and the resulting libraries of mutagenized cells were infected with reovirus serotype 1/Lang at a multiplicity of infection (MOI) of 35, to select for clones resistant to lytic infection. The isolated clones did not express reoviral antigens, and did not produce infectious virus as assessed by plaque assay, suggesting these reovirus resistant clones were not PI.

**Figure 2:**
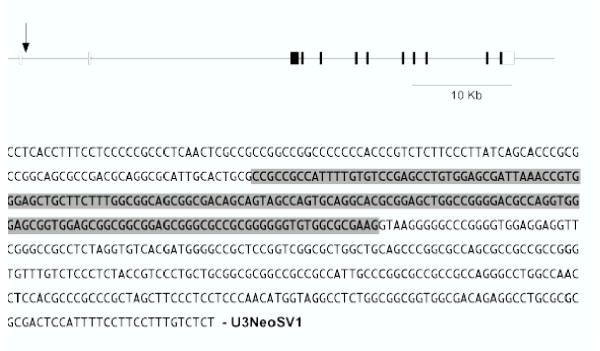
**Disruption of the *Ctcf *gene in 6B72 cells. **DNA sequences flanking the U3NeoSV1 provirus in 6B72 cells were cloned and sequenced. The flanking sequences were identical to sequences in the rat genome, placing the provirus in the first intron of the *Ctcf *gene (A). Filled and open boxes indicate coding and non-coding exons, respectively. Flanking sequences 5' of the U3NeoSV1 provirus (B) include the first exon (shaded) of the gene.

Regions of genomic DNA adjacent to the U3NeoSV1 provirus in each virus resistant clone were isolated by plasmid rescue and sequenced. Altogether, of the 151 isolated clones, 62% of flanking sequences matched known or presumptive genes, and an additional 23% were represented in the public databases of expressed sequence tags (dbEST) or non-redundant sequences (nr). From the 111 clones matching known or presumptive genes, 10 genes were represented more than once. Many of the disrupted genes have known or imputed functions, and several are known to function in the same or related pathways. For example, the library included 4 independent mutations involving three genes that encoded proteins associated with the insulin growth factor-2 (IGF-II) signalling pathway, namely, IGF-ll/manose-6-phosphate receptor [5,6] (*Igf2r*, locus ID 25151), the IGF binding protein 5 protease [7] (*Prss11*, locus ID 65164, 2 clones), and CTCF (*Ctcf*, locus ID 83726), a transcriptional repressor of IGF-II involved in paternal imprinting [17,18]. The position of the provirus (clone 6B72) in the first intron of the *Ctcf *gene is shown in Figure 2. CTCF differentially represses maternal *Igf2 *gene expression, whereas the imprinted paternal gene escapes repression due to methylation of CTCF binding sites [17,18].

### Igf2 transcripts induced by CTCF disruption

**Figure 3:**
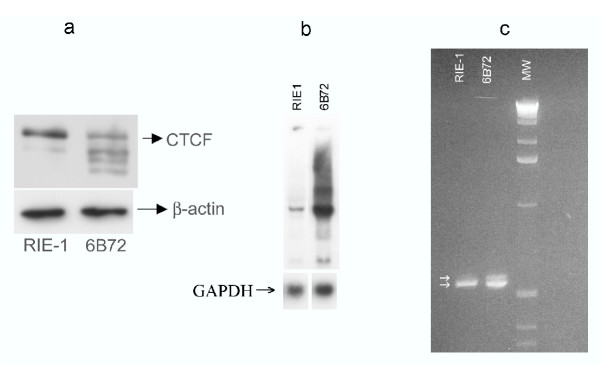
**CTCF and *Igf2 *expression in RIE-1 and 6B72 cells. **Levels of CTCF protein were assessed by Western blot analysis (A), normalized to a β-actin control. Levels of *Igf2 *transcripts were assessed by Northern blot analysis (B) normalized to GAPDH control. Protein content, as assayed by western blot analysis and standardized to β-actin was decreased in the 6B72 cell clone to 30% of control. Reverse transcriptase PCR analysis of *Igf2 *transcripts (C). The RT-PCR products (arrows) were separated on a 2% agarose gel, revealing an additional transcript in the 6B72 cells. The DNA sequence of the larger RT-PCR product (D) revealed an alternatively spliced transcript (*Igf2*^*sv*^) generated by splicing of exon 2 to a cryptic 3' splice site located 14 nucleotides upstream of exon 3.

**Figure 4:**
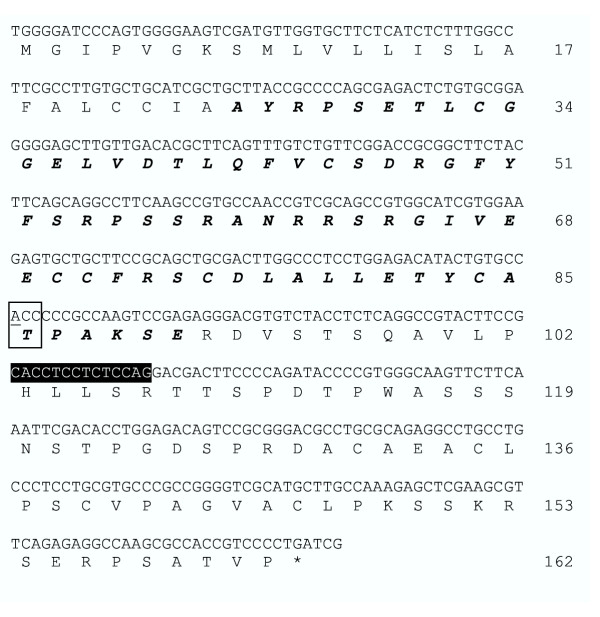
**Alternatively spliced product contains a single nucleotide polymorphism in the igf2 coding sequence. **Intron sequences incorporated into the alternatively spliced transcript (highlighted in black) alter the translational reading frame of the pro-homone downstream of the coding sequence of the processed IGF-II protein (italics and bold). The *Igf2*^*sv *^PCR product also contained a G to A base substitution (underlined) that replaces alanine with threonine at codon 62 (boxed) of the mature hormone.

Considering that four clones had mutations in the IGF-II pathway, the relationship between the *Ctcf *mutation in 6B72 cells and the virus resistant phenotype was investigated further. Levels of CTCF protein in 6B72 cells were reduced by about 50% as assessed by western blot analysis (Figure 3A), consistent with the disruption of one allele. Diminished CTCF expression was associated with an increase in *Igf2 *transcripts as assessed by Northern blot hybridization (Figure 3B). In addition, two products were amplified from 6B72 cells by RT-PCR using primers flanking the IGF-II pro-hormone coding sequence (Figure 3C). The first product was identical to the rat IGF-II pro-hormone (pro-IGF-II) coding sequence (Accession X17012), whereas, the second contained 14 additional nucleotides generated by splicing of exon 2 to an alternative splice acceptor located 14 nucleotides upstream of the normal *Igf2 *exon 3 splice acceptor site. The alternative transcript is expected to encode a pro-IGF-II protein extending 11 amino acids into the E-peptide sequence ending in a stretch of 60 amino acids lacking homology with any known protein (Figure 4).

### Resistance to reovirus lytic infection results from increased Igf2 expression

**Figure 5:**
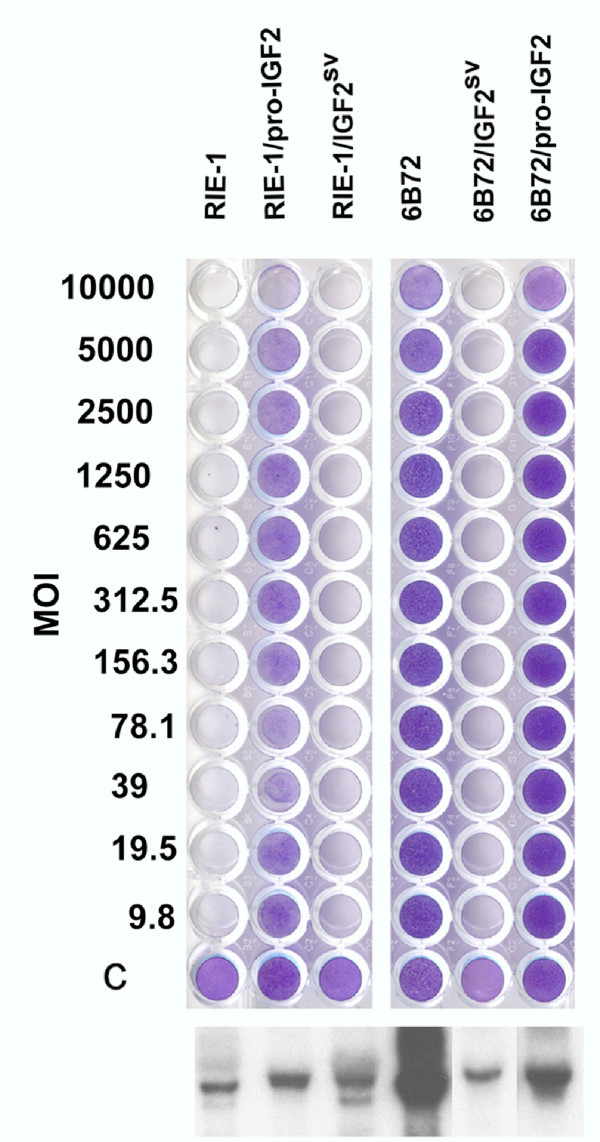
**IGF-II modulates reovirus resistance in RIE-1 and 6B72 cells. **RIE-1 and 6B72 cells expressing either the IGF-II pro-hormone (proIGF2) or the alternatively spliced transcript (IGF2^SV^, Figure 3D) were challenged with serial dilutions of reovirus type 1 (upper panel), and the surviving cells were stained with gentian violet 4 days post-infection. The multiplicity of infection (MOI) for each row is indicated. pro-IGF-II converted RIE-I cells to a reovirus resistant phenotype (RIE-1/proIGF2) but had little if any effect on already-resistant 6B72 cells (6B72/proIGF2). Plasmids expressing the alternatively spliced Igf2 transcript had no effect on RIE-1 cells (RIE-1/IGF2^SV^) but abrogated virus resistance in 6B72 cells (6B72/IGF2^SV^). The experiment was repeated 3 times. Expression of I*gf2 *transgenes (lower panel) was monitored by Northern blot hybridization, and the expression of Igf2 in RIE-1 cells is shown for comparison. Expression of *Igf2 *in 6B72 is not shown.

The stability of virus resistance in the 6B72 cell was tested by infecting RIE-1 and 6B72 cells with reovirus type 1 at a MOI of 10. There was approximately a 10 fold lower titre of reovirus obtained following infection of 6B72 cells as compared to RIE-1 cells at 24 hrs. post-infection (4.5 × 10^5 ^versus 5.1 × 10^6^), and the difference was also maintained at 48 hours post-infection (data not shown). Additionally, there was a dramatic difference in the survival of 6B72 cells after being exposed to high titres of reovirus type 1 (Figure 5).

To determine whether *Igf2 *confers resistance to reovirus infections, clones of RIE-1 cells over-expressing the full-length *Igf2 *transcript or the splice variant (*Igf2*^*sv*^) were generated and examined for their capacity to resist lytic infection. As shown in Figure 5 expression of wild type (*Igf2*), but not the splice variant (*Igf2*^*sv*^) increased the resistance of RIE-1 cells to reovirus infection by over 100 fold. However, when the *Igf2*^*sv*^was transfected into 6B72 cells, the ability of 6B72 cells to survive infection was abolished. Expression of the *Igf2 *gene in an anti-sense orientation caused no significant difference in the capacity of 6B72 cells to resist infection (data not shown). These studies suggest that increased *Igf2 *expression in 6B72 cells is associated with their capacity to resist reovirus infection, and that the *Igf2*^*sv*^encodes a trans-dominant isoform that blocks the activity of *Igf2*.

### The IGF-II hormone confers resistance to lytic infection

**Figure 6:**
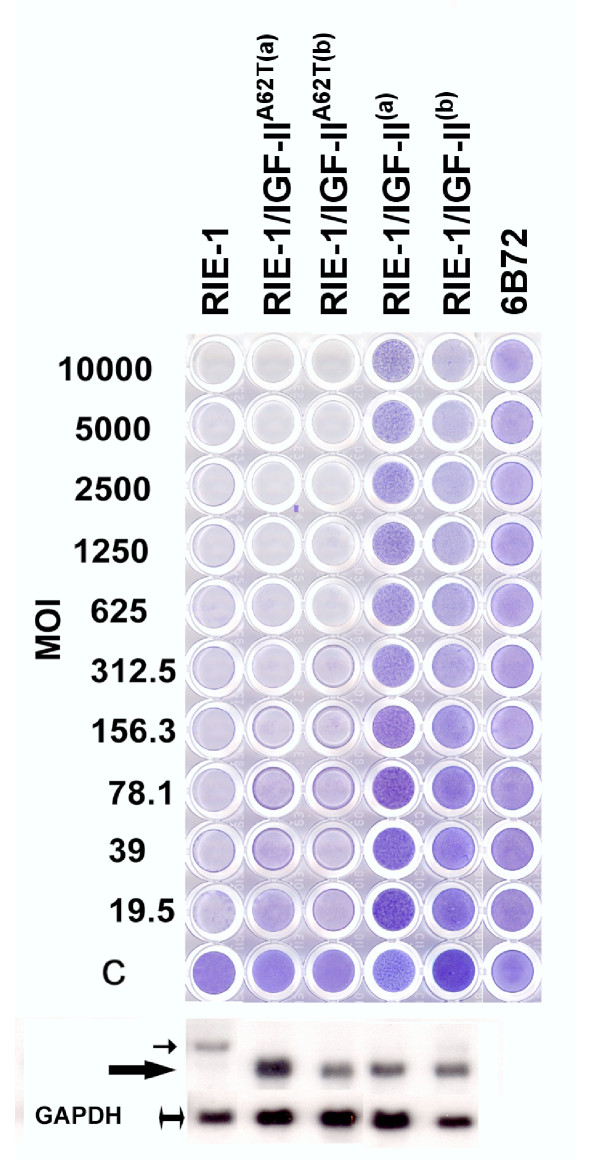
**IGF-II sequences lacking the E-peptide can convert RIE-1 cells to a reovirus-resistant phenotype. **Two independent clones of RIE-1 cells transfected with plasmids expressing native (IGF-II) and mutant (IGF-II^A62T^) proteins without the carboxyl terminal extension-peptide (E-peptide) were challenged with serial dilutions of reovirus type 1 as described in Figure 4. Native IGF-II protected RIE-1 cells from reovirus infection (IGF-II^(a) ^and IGF-II^(b)^), while the IGF-II^A62T ^mutant (IGF-II^A62T(a) ^and IGF-II^A62T(b) ^did not. Non-infected RIE-1 cells (C), and infected 6B72, and RIE-1 cells were included as controls. Expression of *Igf2 *transgenes (lower panel) was monitored by Northern blot hybridization, and the expression of Igf2 in RIE-1 cells is shown for comparison. The native IGF-II (small arrow) is slightly larger than the cDNA constructs (larger arrow), whereas the double-sided arrow marks the constitutively expressed *GAPDH*, as shown. Expression of *Igf2 *in 6B72 is not shown.

*Igf2 *transcripts encode a pro-hormone of 180 amino acids that is processed to generate the 67-residue IGF-II protein [19]. Other proteolytic products including the 89-residue carboxy-terminal E peptide may also be biologically active [20]. The *Igf2*^*sv *^PCR product also contained a single nucleotide substitution (G1393A) that results in the substitution of a threonine for alanine at position 62 of IGF-II, raising questions about *Igf2 *sequences that influence reovirus resistance. Vectors expressing only the 68-residue IGF-II protein both with and without the A62T change were compared for their ability to confer resistance to lytic infection by reovirus type 1 (Figure 6). The IGF-II expression vector converted RIE-1 cells to a virus resistant phenotype; whereas, the IGF-II^62T ^expression plasmid was inactive (Figure 6) and did not suppress the resistance of 6B72 cells (data not shown). These results indicate that virus resistance can be affected by mutations in the IGF-II coding sequence and downstream sequences, including the E-peptide, are not required. However, the trans-dominant effects of *Igf2*^*sv *^apparently require alterations to the carboxy-terminus of the IGF-II pro-hormone.

### Over expression of *igf2 *confers resistance to other reovirus sertotypes

**Figure 7:**
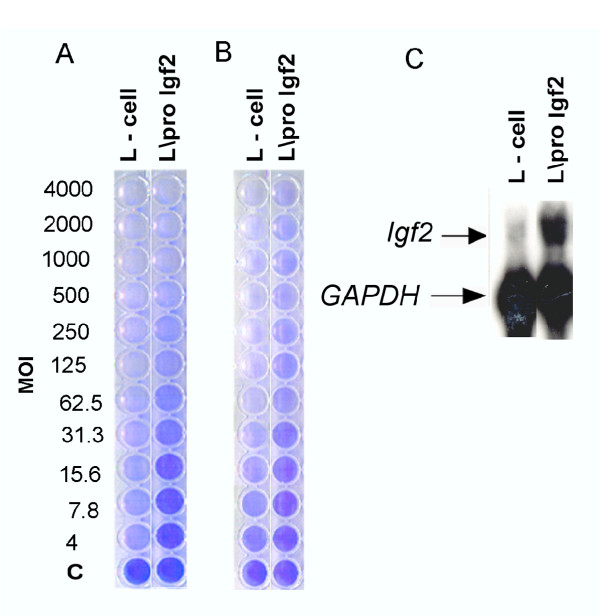
**Decreased lytic infection of L-cell clones over expressing the IGF-II gene. **Constitutively expressed GAPDH was used to assess loading of RNA in lanes. Survival of lytic infection was determined by infecting 10^5 ^L-cells or L-cells expressing the pro-IGF2 transgene with varying concentrations of reovirus type 1 (A) or type 3 (B). The multiplicity of infectious virus particles per cell (MOI) is indicated for each virus serotype. Surviving cells were visualized at 4 (A) or 5 days (B) following infection with gentian violet. Transgene expression was determined by northern blot (C). Experiments were repeated three times and a representative experiment is shown.

RIE-1 cells are intrinsically resistant to infection by reovirus type 3 [13]; therefore, experiments to assess the effects of IGF-II expression on lytic infection by reovirus type 3, were performed using murine L-cells. As shown in Figure 7, L-cells expressing *Igf2 *were significantly more resistant to both reovirus serotypes than the parental cells, indicating that the ability of IGF-II to confer resistance to reovirus infection is not limited to a single cell or virus type.

### IGF-II expressing RIE-1 cell has delayed disassembly of virus particle

**Figure 8:**
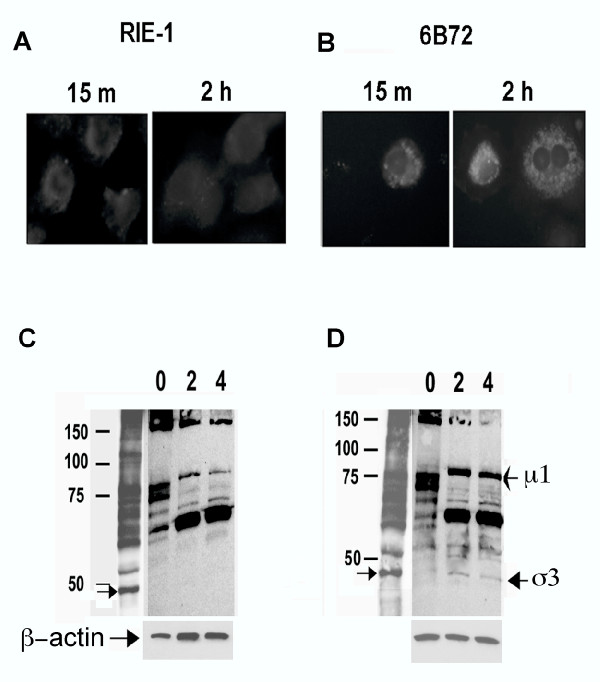
**6B72 cells have delayed disassembly of reovirus type 1. **Fluorescein-labelled reovirus particles were absorbed to RIE-1 (A) or 6B72 cells (B). Persistent fluorescence at 2 hours was found in 6B72 cells, but not in RIE-1 cells. Non-replicating reovirus type 1, at 3 × 10^4 ^particles per cell, was adsorbed to RIE-1 (C) or 6B72 (D) cells at 4°C, washed and incubated at 37°C for 2 and 4 hours. Cells were lysed and the state of virus particles determined by western blot. The outer capsid proteins μ1 and σ3 are present in the 6B72 cell preparations at 2 and 4 hours, but not in the RIE-1 cells.

Previous studies have indicated that proteoytic disassembly of virus particles occurs in the lysosome and requires the participation of cathepsin B and L that are transported by the *Igf2r *gene [21-23]. *Igf2 *binds to *Igf2r *resulting in alteration in cathepsin trafficking [24]. Georgi and colleagues showed that directly fluorescentated reovirus particles dissipated their fluorescent signal coincident with disassembly of the outer capsid [25]. To determine whether over expression of *igf2 *would affect disassembly of virions, we examined the fate of virus during the first hours of infection. Purified reovirus type 1 virions were directly labeled with fluorescein and adsorbed to RIE-1 or 6B72 cells, and the persistence of a fluorescein signal determined at 2 hrs following virus absorption (fig 8A,8B). The binding of virions to RIE-1 cells and 6B72 cells and initial accumulation of virus particles within cells was similar, however fluorescence was almost non-detectable in RIE-1 cells (Figure 8A) whereas it was still present in 6B72 cells at 2 hrs (Figure 8B). In addition, western blot analysis of virion proteins 2 hrs following attachment to cells confirmed the persistence of the σ3 protein in the 6B72 cell clone, but not RIE-1 cells (fig 8C,8D). Therefore, disassembly of virions is altered in the 6B72 cell clone.

### CTCF deficient RIE cells grow in soft agar, a consequence of increased Igf2 expression

**Figure 9:**
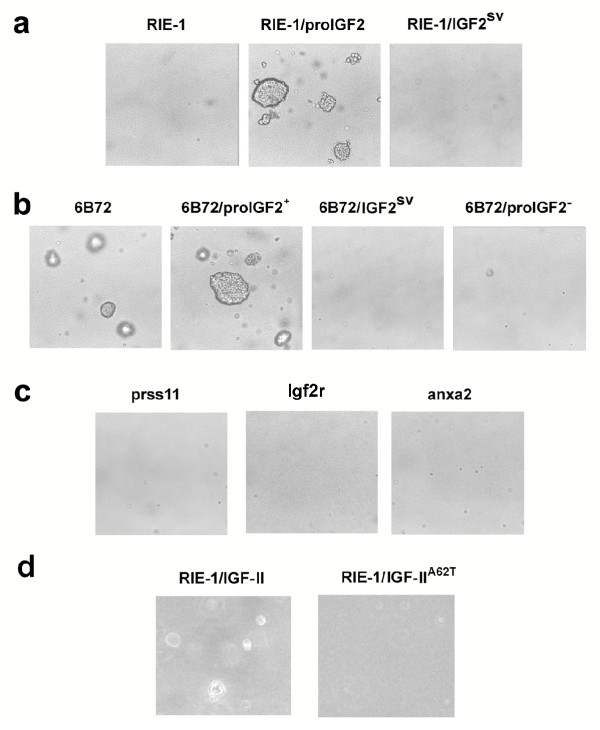
**Anchorage-independent growth phenotypes of RIE-1 cell clones. **10^5 ^cells were suspended in media containing 1% agarose and plated in 6 well culture dishes. RIE-1 cells acquire the ability to grow in soft agar after being transfected with a vector expressing pro-IGF2 but not the IGF2^SV ^splice-variant (a). The vector inserted in the *Ctcf *gene (6B72) confers the ability to grow in soft agar, but the phenotype is suppressed by expression of IGF2^SV ^(b). Clones selected for reovirus resistance with gene trap vectors inserted into the *Prss11*, *Igf2r *and *Anxa2 *genes failed to grow in soft agar (c). RIE-1 cells expressing native IGF-II protein without the E-peptide grew in soft agar but the colonies were smaller (d) than produced by pro-IGF-2 (a), while the corresponding IGF-II^A62T ^protein (E-peptide) did not transform RIE-1 cells to anchorage independence (d). Colonies were photographed (20×) after 7 days except (d) where the cells were photographed after 10 days.

Accumulating evidence suggests that enhanced IGF-II expression associated with loss of *Igf2 *genomic imprinting may promote tumour formation [26,27]. Although RIE-1 and 6B72 cells appeared to proliferate at similar rates as assessed by MTS/PMS incorporation (Data not shown), 6B72 cells that have a disrupted *Ctcf *gene were capable of forming colonies in soft agar (Figure 9b). To assess the role of IGF-II in anchorage-independence, cells transfected with *Igf2 *and *Igf2*^*sv *^were tested for their ability to grow in soft agar. RIE-1 cells transfected with *Igf2*, but not *Igf2*^*sv *^formed colonies in soft agar (Figure 9a), whereas 6B72 cells transfected with *Igf2*^*sv *^lost their capacity to proliferate in soft agar as did cells transfected with an anti-sense *Igf2 *construct (Figure 9b). The IGF-II^62T ^expression plasmid had no transforming activity (Figure 9d). On balance, transforming activities of the different *Igf2 *plasmids on RIE-1 and 6B72 cells as assessed by the soft agar assay were similar to their effects on reovirus resistance, although the vector expressing only IGF-II was less active in transforming RIE-1 cells to anchorage independence than vectors expressing the entire pro-hormone.

**Figure 10:**
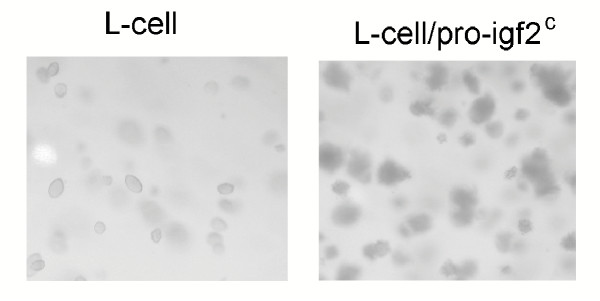
**IGF-II increases colony formation of L-cells in soft agar. **Forced expression of the rat pro-Igf2 gene (pro-Igf2^c^) in L-cells increases the number of soft-agar colonies by 4 to 5 fold as compared to the L-cell parent, as shown in this representative photomicrograph at 7 days (10×).

The capacity to proliferate in soft agar was not a property of other reovirus resistant cell clones that contained mutations in the IGF-II pathway. Figure 9 shows that cell clones with disrupted *Igfr *or *Prss11 *genes did not form colonies in soft agar (Figure 9c). An additional reovirus resistant-clone with an insert in the *Anxa2 *(annexin 2) gene, associated with cytomegalovirus infection [28] and recognized to bind to the insulin and insulin growth factor receptor-1 [29], also failed to grow in soft agar (Figure 9c). Therefore, the capacity to proliferate in soft agar was not a general property of reovirus-resistant cells, even in clones that contain mutations in the IGF-II pathway. However, L-cells also displayed enhanced ability to grow in soft agar in addition to virus resistance, following enforced *Igf2 *expression (Figure 10).

## Discussion

Insertional mutagenesis provides an approach to identify genes associated with selectable cellular phenotypes. We have isolated over 100 potential clones with mutations in genes that may play roles in the life cycle of reovirus. In the present study, one clone resistant to reovirus lytic infection contained a provirus inserted into the gene encoding the CTCF transcriptional regulator. CTCF binding motifs are present in many genes, including *Igf2 *[26], *H19 *[27], and *Myc *[8]. However, since 3 other clones selected for reovirus resistance contained mutations in the IGF-II pathway, the role of IGF-II in virus-resistance was investigated further. Reduced expression of the *Ctcf *gene was associated with enhanced *Igf2 *expression in virus-resistant cells, while forced expression of the *Igf2 *gene in the parental RIE-1 line was sufficient to confer resistance to lytic reovirus infection.

By inference, the recovery of inserts affecting other genes in the IGF-II signalling pathway suggests that mutations in multiple genes may affect the same phenotype by acting on a common pathway. The insert in *Igf2r *was found to decrease the expression of the gene as assayed by northern blot analysis (data not shown). As IGF-II targets the *igf2r *to lysosomal degradation, mutations in the genes encoding either the receptor (*Igf2r*) or its ligand (*Igf2*) will affect the activity of the other, and result in a reduced endosomal trafficking of hydrolases necessary for reovirus disassembly [24]. Our data indicates there is a decrease in virus disassembly in 6B72 cells, consistent with a block at this step in morphogenesis. Further studies will be required to assess if and how inserts in the *Prss11 *and *Igf2r *genes influence reovirus resistance.

As the entry, disassembly, transcription, translation, and repacking of viruses share common features; we anticipate that common cellular pathways will influence infection by other virus families. Indeed, *Igf2r *has been implicated in herpes simplex and zoster virus infection [30,31]. However, the present study is the first to show a direct connection between *Igf2 *gene expression and resistance to lytic virus infection. By using constructs that encode the mature hormone without the E-peptide, we were able to show that forced IGF-II expression is sufficient to confer a reovirus resistant phenotype. These results differ from other studies in which reovirus replication was enhanced by treatment of RIE-1 cells with insulin. The latter effect is presumably caused by enhanced virus replication associated with cell proliferation [13].

Expression of the *Igf2 *gene is frequently elevated in common childhood and adult neoplasms [27,32-38] and has been associated with tumour progression and metastasis [39,40]. *Igf2 *also transformed RIE-1 cells to anchorage-independence, a phenotype that predicts the potential for malignant growth *in vivo*. Virus-resistant 6B72 cells also grew in soft agar, presumably as a result of enhanced IGF-II expression. Since plasmids expressing only IGF-II were less active in transforming RIE-1 cells to anchorage independence than vectors expressing the entire pro-hormone, further study is needed to determine whether the capacity of RIE-1 cells to proliferate in soft agar is enhanced by the E-peptide or other products derived from the carboxy terminus of the pro-hormone. In other studies, the E-peptide enhanced insulin secretion from β-cells [20], and may play a role in cellular transformation [31].

cDNA clones of an alternatively spliced *Igf2 *transcript (*Igf2*^*sv*^), blocked the ability of IGF-II to promote reovirus resistance and anchorage independent growth in a trans-dominant manner. The protein coding sequence of *Igf2*^*sv*^contains a frame shift in the E-peptide region and lacks a site [20,41] required for the proteolytic processing of the pro-hormone. The alternative splice site is used very infrequently [only one EST (AA259833) in dbEST was similarly spliced] and thus probably plays no physiological role. Further studies are planned to determine molecular basis for the dominant negative activity of *Igf2*^*sv*^.

6B72 cells were highly resistant to reovirus infection as determined by virus yield and cell survival at different times post-infection. Virus resistance was a genetically selected trait manifested by clonally pure cell populations, could be conferred by enforced IGF-II expression, and involved decreases in virus disassembly. Although decreased virus disassembly is sufficient to explain virus resistance, we do not exclude the possibility that other mechanisms may contribute to the resistance of 6B72 cells, since any genetic selection may generate clones with multiple, independent mutations. The fact that an early step in infection (uptake/disassembly) is defective in 6B72 cells makes it very difficult to test whether downstream steps might also be affected.

While the original impetus of our studies was to understand the replication cycle of intracellular pathogens that cause acute and chronic infectious diseases, the finding of cell growth phenotypes associated with virus resistance is of some interest. It has been proposed that lytic viruses may used to treat certain malignancies [42-45]. However, based upon our observations, such therapy may carry a risk associated with selection of virus resistant cell clones with enhanced growth/survival potential. Additionally, chronic infections contribute to the development of a number of human cancers [46-49]. While the carcinogenic process is not well understood, cell proliferation associated with inflammation is also thought to contribute to tumour promotion [50,51]. The present study illustrates how carcinogenesis could also be influenced by selection for virus-resistant cells with mutations in genes affecting cell proliferation or survival.

## Conclusions

This is the first reported use of gene entrapment to identify host genes affecting the susceptibility of cells to virus infection. These results indicate that IGF-II, and by inference other components of the IGF-II signaling pathway, can confer high levels of resistance to lytic reovirus infection. IGF-II expression specifically blocked virus disassembly. *Ctcf *disruption and forced *Igf2 *expression both enabled cells to proliferate in soft agar, a phenotype associated with malignant growth *in vivo*. Therefore, these results illustrate a potential indirect mechanism of viral carcinogenesis by which cells selected to virus resistance may also have enhanced oncogenic potential.

## Methods

### Entrapment mutagenesis and selection of reovirus resistant clones

To identify genes required for reovirus lytic infection, a gene trap retrovirus shuttle vector, U3NeoSV1, was used to generate mutagnized rat intestinal (RIE-1) cells [52]. RIE-1 cells were infected with the gene trap vector at a multiplicity of infection <0.1, and were selection in media containing G418 sulfate (0.7 mg/ml) (Clontech, Palo Alto, CA, USA)[52]. Twenty libraries of mutant RIE-1 cells, each consisting of 10^4 ^independent gene entrapment events, were generated and expanded until each mutant clone was represented by approximately 10^3 ^sibling cells. These cells were plated at low density and incubated in serum-free media for 3 days until they became quiescent, infected overnight with reovirus serotype 1 at a multiplicity of infection of 30 plaque forming units (pfu) per cell. The infected cells were detached with trypsin, DMEM medium containing 10% fetal bovine serum (FBS) (Hyclone Laboratories, Inc., Logan, UT, USA) was added and the cells were allowed to reattach. After 4–6 hours the medium was replaced with serum-free medium and the cells were incubated for several days until only a few cells remained attached to the culture flask. Cells that survived the selection were allowed to form colonies that were expanded for further analysis.

### Reovirus stocks and infectivity assays

Reovirus type 1 (Lang) and reovirus type 3 (Dearing) were previously described [53]. A stock of reovirus that was passaged twice in L cells was purified [14] and the purified virus band was fluorescein labelled as previously described [25]. For some experiments the top component, consisting of virus particles that are devoid of genome, was used to study the entry pathway [54].

Survival of parental L- and RIE-1 cells and RIE-1 and L-cells transfected with *Igf2 *constructs was determined in 96-well plates seeded at 5 × 10^4 ^per well. On the following day, serial dilutions of reovirus type 1 or type 3 were added in 100 μl of media and cells were incubated at 37°C and 5%CO_2 _for 1 hour. Cells were washed three times in PBS, and fresh media was added containing 0.1% anti-reovirus antibodies to inhibit secondary infection. Cells were incubated for 4 to 5 days, and surviving cells visualized with gentian violet. Studies were repeated a minimum of three times.

To determine the titre of reovirus present in cells, cells were frozen and thawed three times and plaque assays were performed as previously described [14]. Titres of virus were repeated twice.

### Fluorescence microscopy

For experiments involving fluorescein labeled reovirus, cells were grown on glass slides and fixed following appropriate times with 4% paraformaldehyde, dehydrated, and mounting in cytoseal acrylic resin (Stephens Scientific, Cornwall NJ, USA) to improve clarity and prevent bleaching. Fluorescence microscopy was performed using an Axiophot microscope (Carl Zeiss, Inc., Thornwood, NY, USA), with a 40×/1.3 plan Neofluar objective and fluorescein filter set. Images were captured with a low-light, cooled CCD camera (Micromax, Photometries, Inc., Tucson, AZ, USA).

### Identification of genes disrupted by gene entrapment

To identify the gene disrupted by the vector in clones surviving reovirus infection, the shuttle-vector property of U3NeoSV1 was utilized. Regions of genomic DNA adjacent to the U3NeoSV1 provirus were cloned by plasmid rescue, and sequenced [52]. Sequencing was done using an automated sequencer (ABI 3700 DNA Analyzer, Applied Biosystems, Foster City, CA, USA), and the results obtained were compared to databases available in the public domain (BLAST nr, est, and hgts). The probability of a match to sequences in the databases occurring by chance alone varies due to interspecies conservation and the length of the match. Matches to characterized genes were considered significant if the interspecies matches had a probability score p <10^-5 ^and involved non-repetitive sequences. As indicated, virtually all of the genes identified had matches to murine or human gene sequences with p < 10^-10 ^and rat with p < 10^-20^.

### Igf2 expression was assessed by northern blot hybridization

Total RNA was isolated from cultured cells using Trizole reagent (Gibco BRL, Gaithersburg, MD, USA). 5 μg of RNA was separated on 1.2% agarose gel, and transferred to a nitrocellose membrane. Membranes were hybridized with random prime-labeled (Strategene, Cedar Creek, TX, USA) probes corresponding to a full length of *Igf2 *cDNA and either *glyceraldehyde dehydrogenase (GAPDH*) or β-actin cDNA.

### Igf2 cDNA isolation and expression

Rat *Igf2 *cDNAs were obtained using reverse transcriptase PCR (RT-PCR). Total RNA was extracted from RIE and *6B72 *cells using Trizole reagent (Life Technologies, Rockville, MD, USA). RT was performed on 1 μg of total RNA (PTC-100 programmable Thermal Controller, MJ Research. Inc, Watertown, MA, USA). A pair of primers was designed according to rat sequences: CTTCCAGGTACCAATGGGGATC (forward) and TTTGGTTCACTGATGGTTGCTG (reverse). A 500 bp DNA was amplified under following conditions: 95°C, 1 min; 40 cycles of 95°C 30 seconds, 60°C 30 seconds and 68°C 3 minutes; 68°C 10 min; 4°C.

### Immunoblotting analysis

Cells were washed with PBS and lysed in SDS Lamelli buffer. Protein concentration was determined using the bicinchoninc acid protein assay (Sigma-Aldrich Corp., St. Louis, MO, USA). 20 μg of protein extract was loaded in each lane of a 10% SDS-PAGE and run at 100 V. Protein was transferred to a nitrocellulose membrane at 22 V overnight at 4°C. The membrane was washed three times with TBST (50 mM Tris pH 7.5, 150 mM NaCI, 0.05 % Tween 20) and then incubated in blocking buffer (TBST and 5% non fat dry milk, pH 7.5) for 1 hour at room temperature. The membrane was then incubated with anti-mouse, CTCF (1:500, BD Transduction laboratories) and β-actin (1:3000, Sigma-Aldrich Corp., St. Louis, MO, USA) in blocking buffer overnight at 4°C. Following 3 washes, the membranes were incubated with goat anti-mouse secondary antibody (1:20,000, Jackson ImmunoResearch Laboratories, West Grove, PA, USA) for 1 hour at room temperature, followed by three 15-min washings. Immune complexes were visualized by addition of chemiluminescence reagent (Renaissance, DuPont NEN, Boston, MA, USA) and the membrane was exposed to Kodak XAR-5 film (Eastman Kodak Co., Rochester, NY, USA).

### Transfection

Cells were cultured to semi-confluence and plasmids expressing wild type and variant IGF-II transcripts were transfected into RIE-1 and 6B72 or L-cells using SuperFect Reagent (Qiagen, Inc. Valencia, CA, USA) according to the manufacturer's protocol. After 48 hours, transfected cells were passaged, 1:10, into medium containing hygromicin B (selective medium) at a concentration determined to kill 100% of non-transfected cells (150 mg/ml for RIE-1 and 6B72 cells, 650 mg/ml for L-cells). Cells were maintained in selective medium until clones appeared.

### Soft agar colony forming assay

Dual layers of sea plaque agarose were made with the bottom layer consisting of a 50:50 mixture of 1.6% agarose solution 1:1 and 2X medium. The bottom layer was allowed to set for 4 hours, and then a 50:25:25 solution consisting of 2X medium, 1.6% stock agarose, and 1X medium containing cells, at a final concentration of 5000 cells/ml, was vortexed in a conical tube, and 2 ml was added to each well. Following 30 minutes at room temperature to allow the upper layer to set, plates were incubated at 37°C, 5% CO_2 _incubator for 7–10 days and checked for colony formation by microscopy.

### Analysis of viral protein expression in infected cells

Cells were plated at 1.5 × 10^6 ^per well in 2 ml of medium in 6-well plates and allowed to sit over night. Cells were washed with phosphate-buffered saline (PBS), pH 7.4, and then infected with reovirus type 1 at the specified MOI. Virus was allowed to adsorb to cells for 1.5 hours at 4°C, washed twice with serum-free medium and incubated at 37°C and 5% CO_2_. At the indicated times, cells were scraped and lysed in Tris lysis buffer (10 mM Tris [pH 7.5], 2.5 mM MgCI_2_, 100 mM NaCI, 0.5% Triton x-100, 1 tablet Protease Inhibitor Cocktail Tablets [Roche Applied Science, Indianapolis, IN, USA] per 10 ml). After 30 min on ice, Laemmli sample buffer (Bio-Rad Laboratories, Hercules, CA, USA) were added to cell lysate samples (1:1). Protein samples were loaded in a 12% SDS-PAGE gel and run at 100 V. Protein was transferred to a nitrocellulose membrane at 100 V for 1 hour on ice. The membrane was washed three times with TBST (50 mM Tris pH 7.5, 150 mM NaCI, 0.05 % Tween 20) and then incubated in blocking buffer (TBST and 5% non fat dry milk, pH 7.5) for 1 hour at room temperature. The membranes were then incubated with rabbit anti-reovirus type 1 (1:50) and β-actin (1:3000, Sigma-Aldrich Corp., St. Louis, MO, USA) antibodies in blocking buffer overnight at 4°C. Following 3 washes in TBST, the membranes were incubated with goat anti-rabbit (for reovirus) or goat anti-mouse (β-actin) secondary antibodies (1:20,000, Jackson ImmunoResearch Laboratories, West Grove, PA, USA) for 1 hour at room temperature, followed by three 15-min washes. Immune complexes were visualized by addition of chemiluminescence reagent (Renaissance, DuPont NEN, Boston, MA, USA) and the membrane was exposed to Kodak XAR-5 film (Eastman Kodak Co., Rochester, NY, USA).

### Cell Proliferation Assay

RIE-1, 6B72, or *Igf2 *transfected RIE-1 or 6B72 cells were seeded at 5 × 10^4 ^per well in 96-well plates, incubated at 37°C and 5% CO_2_. At 4, 6, 18, 48 hours post plating, 3-(4,5-dimethylthiazol-2-yl)-5-(3-carboxymethoxyphenyl)-2-(4-sulfophenyl)-2H-tetrazolium [MTS], and an electron coupling reagent (phenazine methosulfate, [PMS]) were added at 20 μl per well (CellTiter 96 Aqueous Non-Radioactiver Cell Proliferation Assay, Promega, Madison, Wl). Plates were incubated for 2 hours, and then the absorbance was determined at 490 nm. Each set of conditions was repeated in triplicate.

## Authors' contributions

JS, ELO, and CH conducted most of the laboratory work. KSW assisted in the analysis of fluorescein-labelled virus preparations. HER provided the vectors and advice on their use. DHR discovered that persistently infected cells require serum to survive, allowing the selection of genetically resistant cell clones. HER and DHR provided funding and supervision for the research, and prepared the manuscript. All authors have read and approved the final manuscript.
